# Interdisciplinary science for future governance and management of forests

**DOI:** 10.1007/s13280-015-0743-8

**Published:** 2016-01-07

**Authors:** Annika Nordin, Camilla Sandström

**Affiliations:** SLU, 901 83 Umeå, Sweden; Department of Political Science, Umeå University, 901 87 Umeå, Sweden

**Keywords:** Forests, Governance, Management, Interdisciplinary science, Actor participation, Societal impact

## Abstract

The sustainable use of forests constitutes one of the great challenges for the future due to forests’ large spatial coverage, long-term planning horizons and inclusion of many ecosystem services. The mission of the *Future Forests* programme is to provide a scientifically robust knowledge base for sustainable governance and management of forests preparing for a future characterized by globalization and climate change. In this introduction to the Special Issue, we describe the interdisciplinary science approach developed in close collaboration with actors in the *Future Forests* programme, and discuss the potential impacts of this science on society. In addition, we introduce the 13 scientific articles and present results produced by the programme.

## Introduction

In Sweden, nearly 70 % of the land area is covered by forest. Forest owners manage approximately 75 % of the forestland for multiple purposes. Private forest owners (ca. 250 000 persons) with relatively small forest holdings own about half of the forestland. Forest companies, the government, municipalities and parishes own the remaining part. All owners are required to manage their forest according to the Forestry Act, which states that forest management should give equal weight to forests’ economic and environmental values. Hence, all forest owners are obliged to sustain wood production while at the same time conserving biodiversity, enhancing recreational needs, protecting waters and soils and mitigating climate change. Due to the multiple layers of property rights (e.g. hunting rights, public right of access “allemansrätten” and reindeer-herding rights held by the Indigenous Sámi population) in parallel to private ownership of the forests, there are a number of competing demands on the forest resource. The economic value of Swedish forestry has been recognized for more than a century, while the forests’ non-market values along with the preservation of its ecological and social values are attracting increasing interest from stakeholder organizations and the public (Sandström and Sténs 2015).

The ongoing processes of globalization and climate change will cause fundamental changes to the present conditions for managing forests in Sweden and elsewhere (Katila et al. 2014). Societies closely interlinked with forests in social–ecological systems will be challenged not only by new opportunities, but also by the increasing uncertainty and risk associated with the long-term planning horizon of forest management. In addition, the increasing and often conflicting demands put on forests by various stakeholders call for decisions on trade-offs between competing interests to be made (Beland Lindahl et al. 2015).

Policy decisions under these circumstances are complex processes as they encompass both social and natural systems. Hence, science-based decision support needs to be interdisciplinary, straddling the social–natural divide (Lélé and Kurien 2011). Under the *Future Forests* (www.futureforests.se) programme hosted by the Swedish University of Agricultural Sciences, and in collaboration with Umeå University and the Forestry Research Institute of Sweden, we have developed an interdisciplinary research environment closely connected to stakeholders, with the purpose of delivering integrated knowledge conducive to provide solutions to the complex problems of future forest governance and management. The 13 scientific articles in this Special Issue form an important delivery from the programme to achieve this overall objective. Its aim is to provide an overview of research related to current and future issues, which is needed to enable an increased and sustainable provision of ecosystem services from boreal forest landscapes in Sweden and elsewhere. We begin by providing an overview of the development and organization of the interdisciplinary and actor-oriented research in *Future Forests*. We then introduce the scientific articles presenting results produced by the programme, and discuss the potential impact of this research on society.

## Stakeholder participation and interdisciplinary science organization in the future forests programme

The start of *Future Forests* in 2009 was an outcome of preparative and agenda-setting activities by a coalition of scientists, non-governmental environmental and recreational organizations, industry, forest owners’ associations and government agencies. This engagement of multiple stakeholders laid a solid foundation for the programme’s societal outreach and subsequent impacts. The programme’s organizational structure was set to ensure stakeholders’ continued involvement via a programme board and topic-specific reference groups.

The broad support from the various stakeholder organizations requires a credible approach to the programme’s communications. Our target groups for communication are the public with an interest in forests, forest owners, officials of relevant forest governmental agencies and ministries, as well as officials of forest companies, non-governmental environmental organizations and outdoor recreation groups. To reach these different stakeholders, we use an array of communication activities: the magazine *Skog och Framtid* distributed twice a year to Sweden’s 250 000 private forest owners, several forest excursions each year and numerous meetings across the country, teaching of undergraduate and graduate studies as well as stakeholder involvement in participatory and transdisciplinary research activities.

Currently the researchers are organized around three main topical themes: *“Biological diversity in future forests: governance and management that combine forest biomass production and nature conservation”, “Waters and soils in future forests: reduced impact from forestry by new approaches in governance, management and practical tools”* and *“Climate change in future forests: governance and management to integrate tools for mitigation and adaptation”*.

## An introduction to the articles in this special issue

The scientific articles in this Special Issue are all linked to the overarching aim to deliver integrated knowledge conducive to provide solutions to the complex problems of future forest governance and management. They are, however, underpinned by different perspectives and depart from different parts of the social and/or ecological system and can thus be grouped into four broad themes: defining forest values in the past and present, considerations of forest values under different forest management systems, developing tools to improve the governance and management of forest soils and waters, and finally climate-change impacts on forestry and forest health.

The three papers in the first group have in common that they examine stakeholders’ target images for the forest and explore potential consequences of these images on the governance and management of forests; the three papers in the second group all address alternatives to today’s dominant methods for forest management and explore the consequences of applying such alternatives; the four papers in the third group focus on forestry’s effects on soils and waters in a landscape perspective; and the three papers in the fourth group study forest’s carbon balances and climate change induced potential serious damage on forests by exotic pests and pathogens.

### Defining forest values in the past and present

Conflicting perspectives on forests challenge the development of forest governance and management in Sweden. Research can explain the bases for disagreements among stakeholders in the past as well as present, explore alternative pathways to move beyond contemporary deadlocks and suggest processes that may lead to acceptance of change. Mårald et al. (2016) explore to what extent ideas or values in forestry represent something new in the ongoing debate. They examine change over time concerning dominant concepts used in Nordic and United States forestry journals in the early twentieth and early twenty-first centuries, and conclude that over time, many competing claims were placed on the forest and that “(F)orests today have multiple meanings and multiple uses to broad publics—and the same was true over a century ago”. Sténs et al. (2016) examine different perceptions between stakeholders regarding forests’ social values and what governance modes and management tools they accept. Although, as expected, tourism and recreation is the most commonly defined concept, the study reveals a great variety of perceptions as well as favoured governance modes and management measures of forest social values. Divergent perceptions of forest values are also prominent in the study of desired forest futures by Sandström et al. (2016). Both papers identify a clear divide between stakeholders that advocate a forest governance model characterized by forest owners’ voluntarism and stakeholders that advocate stronger top-down regulations.

### Considering multiple forest values under different forest management systems

In the Nordic countries, the dominant forest management system applied on the stand level is tree harvest by clear-felling followed by plantation of seedlings and pre-commercial and commercial thinnings. Environmental considerations targeting the conservation of biodiversity are taken mainly at clear-felling by green tree retention and dead wood preservation. Research can suggest alternatives to this dominant forest management system and explore consequences of such alternatives on forests’ economical, ecological and social values. Roberge et al. (2016) explore consequences of varying rotation lengths on forests’ different values. They apply a qualitative approach analysing a large range of ecosystem services to conclude that extending rotation lengths positively affect forests’ delivery of most of these. Felton et al. (2016) assess the effects of mixed tree species forestry on forests’ delivery of ecosystem services. They highlight positive effects of mixed tree species on biodiversity, water quality, aesthetic and recreation values and reduced vulnerability to pest and pathogen damage. The authors, however, find that diversifying forest management carries risks and uncertainties and calls for “comprehensive interdisciplinary evaluations when assessing pros and cons of (tree species) mixtures”. To handle the increasing demand for alternatives to Sweden’s dominant silvicultural system, while at the same time managing uncertainties and risks, Rist et al. (2016) suggest an operational model for adaptive forest management with the purpose of engaging forest owners and managers in introducing new silvicultural systems in Swedish forestry. Their study identifies how “pitfalls” associated with the implementation of adaptive management can be avoided and what is needed in terms of “investments, infrastructure and other considerations” providing what the authors define as a “new paradigm” of adaptive management (Rist et al. 2016).

### Developing tools to improve the governance and management of forest soils and water

The quality of forest waters and soils is tightly coupled to forest growth and the overall functionality of forested landscapes. Climate change, long-range, air-borne pollutants and forestry all cause pressure on forest waters and soils. Research can elucidate mitigation options for these negative pressures. Management strategies need to be applied on a landscape level by combining biogeochemical knowledge with applicable tools for governance and management. Laudon et al. (2016) suggest operationalization to minimize water-quality impacts from forestry via a novel “hydromapping tool” to be applied on the landscape level. Eklöf et al. (2016) address the problem of forestry causing mercury previously deposited over forest landscapes to end up in fish. They conclude that current recommendations in forestry to avoid the mobilization of deposited mercury is sufficient, but that policymakers need to better recognize the complexity of this issue. Sponseller et al. (2016) review current knowledge on links between landscape nitrogen cycling and forest management, particularly dealing with the nitrogen limitation to tree growth. Futter et al. (2016) present a conceptual framework for ranking and communicating water-quality issues in managed landscapes which potentially could assist in implementation of the “polluter pays” principle.

### Forestry mitigating climate change and its impacts on forests

Climate change and related policy measures will cause fundamental changes to the present conditions for managing forests. Alternatives to the predominant even-aged silvicultural system presently practised in Sweden have been studied and analysed from different perspectives. Lundmark et al. (2016) target the practice of continuous cover forestry and compare the carbon balances of this practice with the carbon balance of clear-cut forestry. They apply a set of models to conclude that the greatest climate benefit from forestry results from societal substitution of fossil energy and materials with wood. Hence, the forest management method has only minor influence on the overall carbon balance. Another strand of research related to climate change is the evaluation of the risk for increased damage on forest by pests and pathogens. Research can display ecosystem-associated risks to climate change, but also how these may be underpinned by legislative frameworks. Furthermore, research may appoint potential pathways to deal with the risks. For example, Pettersson et al. (2016) point out that within the EU countries, preventive actions towards trade of plant material that may carry pests and pathogens endangering forest health in receiving countries are strictly limited by free trade agreements. Pettersson et al. (2016) suggest that the Swedish precautionary principle may, however, be used by policy makers to better restrict such risks. Klapwijk et al. (2016) envision a combination of changes in legislation, targeted management with a specific focus on trade commodities and pathways, and processes where the public may be engaged in the battlement against spread of harmful pests and pathogens.

## Concluding remarks

In this Special Issue, we have identified how the multiple values associated with forests are linked to visions of desired future forests among key stakeholder groups in Sweden. We have also explored how various ecosystem services may be affected by changes in silviculture, e.g. changed rotation lengths, mixed tree species forestry, and continuous cover forestry. What can we possibly win or lose by making changes in forest policy and management practices in terms of impact on ecosystem services, and subsequently, the fundamental values relating to the forest? From a societal perspective, these types of interdisciplinary studies analysing and explaining the underlying construction of fundamental aspects of forest values or the studies assessing the impacts on forests’ production and environmental values of management changes play an important role as a basis for future decisions on key aspects in the Swedish forest policy.

We have also identified gaps, e.g. lack of effective policies and institutions to deal with not only wicked problems such as mercury contamination in relation to forestry operations but also the possibilities to implement legislative control to reduce the risk of spreading pest and pathogens. The societal impact of interdisciplinary policy and institutional analysis of this kind, which identify gaps in current policies and legislative frameworks, play an important role in suggesting ways forward on how to bridge the gaps and thus providing important input into current policy processes.

We also presented a number of models and tools that may be used in relation to forest management operations. The pragmatic operational model for adaptive management with the purpose to manage uncertainties and risk associated with silvicultural systems and methods is one of them. Adaptive management has proven to be a useful tool in, for example, wildlife management but has so far rarely been used in relation to forestry. With the suggested model, there is a possibility to more thoroughly examine alternatives to today’s dominant forest management system and address uncertainties through adaptive and flexible management that keep options for the future open. In relation to water-quality issues and forest practices, we have suggested frameworks and tools to rank and communicate water-quality issues and planning instruments to minimize water-quality impacts during forestry operations. From a societal perspective, both framework and tools may provide important input to stakeholders during management processes to set relevant objectives, make appropriate decisions, take measures, and monitor and evaluate the decisions.

In summary, this Special Issue forms one important delivery from the *Future Forests* programme. It illustrates the process of integrating various disciplines into interdisciplinary collaboration. Also it shows that when an interdisciplinary research environment successfully has been established, the research team may produce excellent research with significant societal impact. In this case, the results of the studies may play an important role in the different phases of forest policy and decision-making processes in the Swedish society.
